# Atypical Presentation of Bacteremic Urinary Tract Infection in Older Patients: Frequency and Prognostic Impact

**DOI:** 10.3390/diagnostics11030523

**Published:** 2021-03-15

**Authors:** Caroline Laborde, Julien Bador, Arthur Hacquin, Jérémy Barben, Sophie Putot, Patrick Manckoundia, Alain Putot

**Affiliations:** 1 Médecine Interne Gériatrie, Centre Hospitalier Universitaire Dijon Bourgogne, 21000 Dijon, France; caroline.laborde@chu-nimes.fr (C.L.); arthur.hacquin@chu-dijon.fr (A.H.); jeremy.barben@chu-dijon.fr (J.B.); sophie.putot@chu-dijon.fr (S.P.); patrick.manckoundia@chu-dijon.fr (P.M.); 2 Laboratoire de Microbiologie, Centre Hospitalier Universitaire Dijon Bourgogne, 21000 Dijon, France; julien.bador@chu-dijon.fr

**Keywords:** urinary tract infection, aged, bacteremia, symptoms, fever, mortality, atypical presentation, bacteriuria, immunosenescence

## Abstract

In older patients, urinary tract infection (UTI) often has an atypical clinical presentation, making its diagnosis difficult. We aimed to describe the clinical presentation in older inpatients with UTI-related bacteremia and to determine the prognostic impact of atypical presentation. This cohort study included all consecutive patients older than 75 years hospitalized in a university hospital in 2019 with a UTI-related gram-negative bacillus (GNB) bacteremia, defined by blood and urine cultures positive for the same GNB, and followed up for 90 days. Patients with typical symptoms of UTI were compared to patients with atypical forms. Among 3865 inpatients over 75 with GNB-positive urine culture over the inclusion period, 105 patients (2.7%) with bacteremic UTI were included (mean age 85.3 ± 5.9, 61.9% female). Among them, UTI symptoms were reported in only 38 patients (36.2%) and 44 patients (41.9%) had no fever on initial management. Initial diagnosis of UTI was made in only 58% of patient. Mortality at 90 days was 23.6%. After adjustment for confounders, hyperthermia (HR = 0.37; IC95 (0.14–0.97)) and early UTI diagnosis (HR = 0.35; IC95 (0.13–0.94)) were associated with lower mortality, while UTI symptoms were not associated with prognosis. In conclusion, only one third of older patients with UTI developing bacteremia had UTI symptoms. However, early UTI diagnosis was associated with better survival.

## 1. Introduction

Urinary tract infections (UTI) are the most common source of bacteremia in older patients [1], with a prevalence twenty times higher than in younger patients [2]. However, these frequent infections have to be distinguished from even more common urinary tract colonization in order to avoid abusive and potentially deleterious antibiotic treatment [3]. A careful clinical search for functional urinary signs or symptoms is, therefore, essential, because the mere presence of pyuria or bacteriuria on urinalysis does not allow the clinician to distinguish a true UTI from urine colonization [4]. On the other hand, the literature reports a greater frequency of atypical semiology of infectious diseases in older patients, and particularly atypical UTI [5,6]. The diagnosis of sepsis and its origin are, therefore, more difficult to determine [2,7,8,9,10]. Recently, these atypical presentations have been associated with an increased mortality in bloodstream infections [9].

Geriatricians are aware of these age-related semiologic atypia: facing an aspecific presentation of acute illness, they frequently prescribe microbiological sampling to rule out an acute infection, even in the absence of sepsis criteria. While such procedures frequently result in the identification of bacteriuria/pyuria of clinically undetermined significance [11], they are of particular interest when blood and urine cultures are positive to the same germ Gram-negative bacillus (GNB), as they signal UTI-related bacteremia, even in the absence of symptoms. The Centre of Disease Control has recently recognized this entity as “asymptomatic bacteremic UTI” [12]. However, the frequency and prognosis of this entity remain, to date, undetermined.

In this study, we aimed to (a) describe the clinical presentation in an older population with UTI-related bacteremia, and (b) determine the impact of atypical presentation on the diagnosis, initial management, and prognosis of these patients.

## 2. Materials and Methods

### 2.1. Population

In this retrospective, observational, monocentric study, we included all patients older than 75 years and hospitalized at the Dijon university hospital between 1 January 2018 and 1 January 2019 who presented gram-negative bacillus (GNB) bacteremia with concomitant bacteriuria with the same germ on urinalysis. The criteria of significance for urinalysis were selected according to the current recommendations [5]. Patients were screened using the bacteriology laboratory database.

### 2.2. Data Collection

Socio-demographic data, comorbidities (evaluated with the Charlson comorbidity index) [13], dependence (evaluated with the Katz activities of daily living scale) [14], history of major neurocognitive disorder, polymedication (≥5 drugs), and severe malnutrition [15], were prospectively collected at inclusion. Clinical presentation, site of UTI acquisition (community, nursing home or hospital), biological sampling including C reactive protein and leucocyte count, and urinalysis results were reported at the acute phase of UTI (i.e., at the time of blood culture collection). The initial diagnosis and antibiotic treatment within 24 h, following sampling for blood cultures, was recorded. The following antibiotic treatments were defined as covering the GNB spectrum [5]: third-generation cephalosporins, piperacillin-tazobactam, aztreonam, carbapenemes, fluoroquinolones.

Typical presentations were defined as follows:

-Sign of sepsis: hyperthermia greater than or equal to 38.3 °C [16], and/or hypothermia ≤36 °C, and/or hypotension (systolic blood pressure (SBP) ≤ 100 mmHg), and/or description of chills. The Quick Sequential Organ Failure Assessment (qSOFA) score was used to assess the severity of sepsis;

-Symptoms of UTI: New onset of pain in the lumbar fossa and/or urinary functional signs (urinary burning, urinary frequency, incontinence, suprapubic pain, hematuria) and/or the presence of acute urine retention.

Vital status was recorded by telephone call to the patient (or his/her relative when unavailable) 90 days after the collection of blood cultures.

This observational study was approved by the local ethics committee.

### 2.3. Statistical Analyses

Patients who had a typical presentation of UTI were compared with patients who had no typical signs or symptoms. 

Qualitative variables were expressed as numbers and percentages and compared with the Chi-2 or Fischer tests, as appropriate. Continuous variables were expressed as means and standard deviations and compared with the Student’s *t*-test. 

Kaplan-Meier curves and log rank tests were used to compare survival times according to predetermined parameters: symptoms of bacteremia, symptoms of UTI, hyperthermia, acute urine retention, *Escherichia coli* bacteremia, initial antibiotic therapy. 

Factors associated with mortality at 90 days were analyzed with a backward multivariate analysis by a Cox model integrating clinically relevant variables and/or variables associated with mortality in univariate analysis (*p* < 0.05): age, sex, Charlson Comorbidity Index, qSOFA, temperature, acute urine retention, initial diagnosis of UTI, antibiotics started within 24 h. The threshold for significance was set to 5%. SPSS version 12.0.1 (IBM, Armonk, NY, USA) was used for all statistical tests.

## 3. Results

### 3.1. Population

**Figure 1:**
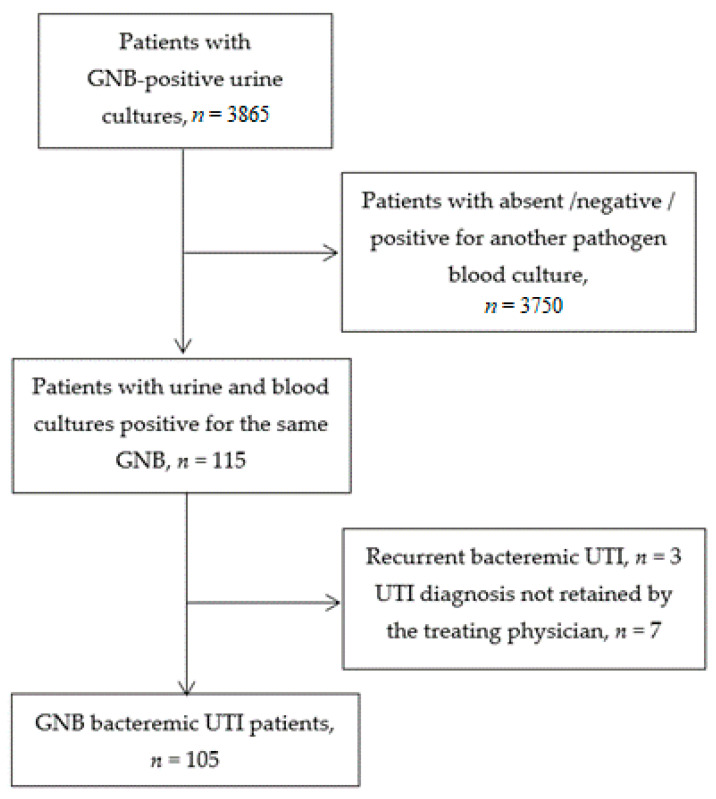
Flow chart (GNB: Gram negative bacillus; UTI: Urinary tract infection).

Among 3865 consecutive inpatients older than 75 years and hospitalized in 2019, with a urine culture positive for a GNB, we included 105 (2.7%) patients with at least one blood culture positive for the same pathogen (Figure 1). Mean age was 85.3 ± 5.9 years and there were 65 women (61.9%). Socio-demographic characteristics and clinical presentation are described in Table 1. 

At the time of blood culture collection, 85 patients (81%) showed signs of sepsis, including 61 febrile patients (59.8%). Only 38 patients (36.2%) had signs or symptoms of UTI. For 90 patients (85.7%), the diagnosis of sepsis was made within 24 h, following blood culture sampling. Sepsis of urinary origin was retained for 64 patients (58.1%). At 90 days, 16 patients (15.2%) were lost to follow-up. Of the patients followed up at 3 months, 21 patients (23.6%) had died. 

### 3.2. Factors Associated with UTI Symptoms

In this older population with UTI-related bacteremia, age was not significantly associated with UTI symptoms. Women were markedly less represented in the group of patients with versus without UTI symptoms (44.7 vs. 71.6%, *p* = 0.01), contrary to patients with a chronic urinary catheter (28.9% vs. 11.9% respectively, *p* = 0.03). Diabetic patients tend to be less frequent in patients with versus without UTI symptoms (15.8 vs. 31.3%, *p* = 0.08). Signs of sepsis (hypotension, chills, fever) were equally present in the two groups, and the qSOFA score did not significantly differ. Unlike the C-reactive protein rate, the leucocyte count was significantly higher in the case of UTI symptoms (14.7 10^9^/l [11.4–19.6] vs. 10.8 10^9^/l (7.6–16.2), *p* = 0.02).

As expected, the diagnosis of UTI-related sepsis was more frequent in the presence of UTI symptoms (81.6% vs. 44.8%, *p* < 0.01) and antibiotic therapy targeting GNB was also more frequently introduced within 24 h (81.6% vs. 62.7%, *p* = 0.04).

### 3.3. Survival Analyses

Factors associated with 90 days mortality are presented in Table 2. 

**Figure 2:**
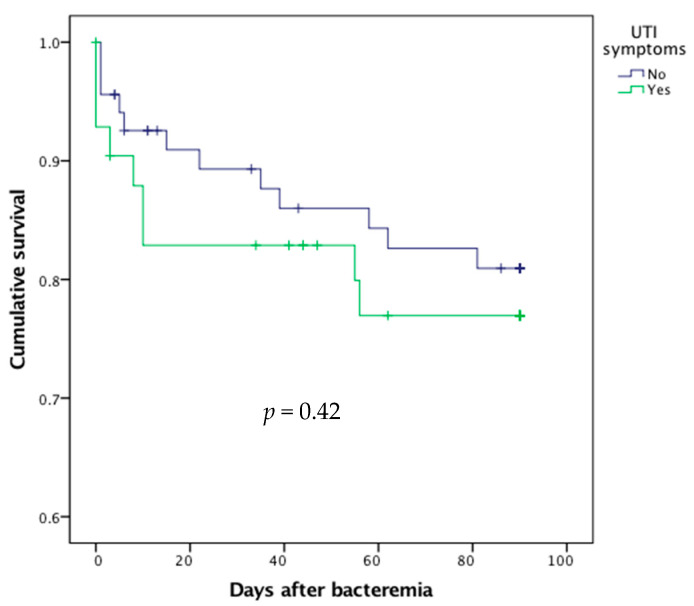
Survival curves after bacteremic urinary tract infection (UTI) according to the presence or absence of UTI symptoms.

In univariate analysis, there was no difference between symptomatic and asymptomatic UTI patients in terms of 90-day mortality rate (*p* = 0.42, Table 2, Figure 2).

**Figure 3:**
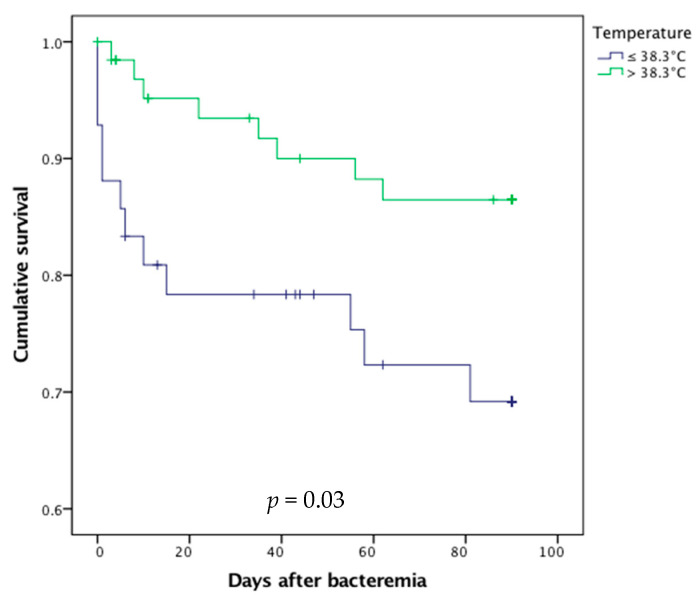
Survival curves after bacteremic urinary tract infection in febrile and afebrile older patients.

Conversely, the absence of fever (i.e., temperature < 38.3 °C, *p* = 0.03, Figure 3) and the presence of acute urine retention (*p* = 0.01) at blood culture sampling were associated with higher mortality.

**Figure 4:**
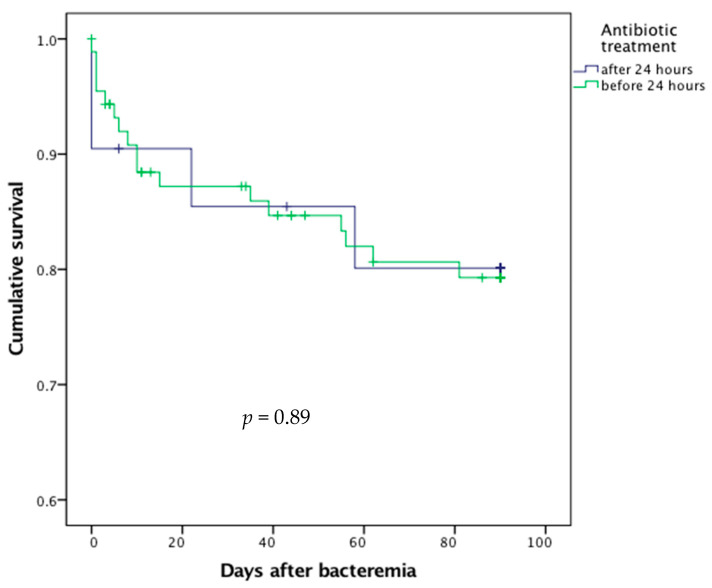
Survival curves after bacteremic urinary tract infection according to the delay in antibiotic treatment.

Delayed prescription of antibiotic therapy did not significantly result in excess mortality (*p* = 0.89, Figure 4).

In multivariate analysis (Table 2), female sex (HR = 0.29; IC95 (0.10–0.79)), hyperthermia (HR = 0.37; IC95 [0.14–0.97]), acute urine retention (HR = 4.29; IC95 (1.41–13.05)) and early diagnosis of UTI (HR = 0.35; IC95 (0.13–0.94)) were associated with lower risk of death at 90 days.

## 4. Discussion

The diagnosis of UTI is a major issue in geriatrics. It is one of the most common diagnostic errors [17] and the leading cause of inappropriate antibiotic therapy in the geriatric setting [18]. The most common pitfall is confounding urinary tract colonization and infection. Indeed, urinary colonization in the very elderly is common, affecting 25–50% of women and 15–40% of men living in nursing homes [5]. The diagnosis of UTI in young patients is based on the fact that urine is normally sterile in a normal bladder. However, in older patients, there is a progressive functional deterioration of vesical function, leading to urinary stasis and explaining the occurrence of significant bacteriuria without any symptoms [3]. The diagnosis of a UTI is, therefore, based, above all, on a clinical evaluation, which is often non-specific. The opposite pitfall is the under-diagnosis of UTI in its atypical forms, which are suspected to be frequent in geriatric medicine but still under-evaluated in the literature. The major issue in practice as well as in clinical research is the absence of a gold standard to distinguish UTI from colonization. To avoid false positives, this study, therefore, focused only on bacteremic forms, for which the diagnosis of UTI is certain.

Given the high uncertainty in UTI diagnosis in this older population, we aimed to evaluate the frequency and prognostic burden of atypical presentation in an unselected cohort of older inpatients with UTI-related bacteremia. The main results are as follows: (1) UTI symptoms were found in only one-third of patients and were not associated with prognosis; (2) Conversely, apyrexia, found in 40% of patients, was associated with a higher risk of death; (3) Early UTI diagnosis was made in 58% of patients and associated with a better prognosis.

This work highlights the low prevalence of UTI symptoms in bacteremic UTI, reported in only one-third of older inpatients, even though these are clinically decisive elements for the diagnosis of UTI-related sepsis. The low effectiveness of UTI signs has already been described: Woodford et al. reported the absence of UTI symptoms in half of an older population with UTI-related bacteremia in comparison to one-fifth of patients under 75 years of age [19]. Other studies confirm that only half of aged patients hospitalized for UTI have urinary symptoms [20,21,22,23,24]. Different hypotheses can be put forward to explain this low proportion. First, the semiology of UTI in the very elderly subject loses its specificity with age [3,5,6], especially in the case of altered mental status [24], even in the bacteremic forms corresponding to parenchymal UTI (pyelonephritis or prostatitis). Second, we may also hypothesize that bacteremia could occur when there is a breakdown of the urothelium barrier and/or translocation from the colonized urinary tract without parenchymal UTI, according to a model already described for the digestive tract [25]. This hypothesis is encouraged by the fact that urine retention and a history of urological surgery are known risk factors for bacteremia [26].

If neither the clinical signs nor the results of urinalysis are sufficient to make a definite diagnosis of UTI, how can we improve our diagnostic skills? The criteria developed by McGeer and Loeb for this purpose have a limited performance [5,27,28]. Several biomarkers have been discussed in recent years, but, to our knowledge, none have been validated in routine practice [29]. Among them, urinary interleukin 6 level appears to be higher in elderly patients with acute UTIs than in patients with asymptomatic bacteriuria [30,31,32]. Conversely, a recent study in nursing home residents highlights that serum inflammatory biomarkers, namely C reactive protein and procalcitonin, are not suitable tests for distinguishing UTI and asymptomatic bacteriuria [33]. Finally, since the sensitivity and specificity of blood cultures are not influenced by age [8], this test remains of interest and should not be overlooked in the case of atypical symptoms.

It is now well-established that older patients frequently present, in a septic context, atypical or non-specific symptoms [2,6,7,8]. Nearly two-thirds of patients over 85 years of age are reported to have atypical symptoms in bacteremia [7]. Impaired instrumental ability of daily living is thought to be a predictive sign of UTI [22]. These atypical presentations are well known by clinicians and motivate the prescription of urinalysis in patients with unexplained functional decline, at the cost of potentially unnecessarily prescribing antibiotics.

As expected, the diagnosis of sepsis was retained in our study for the majority of patients for whom blood cultures were taken, and antibiotic therapy was started for 80% of patients at initial management, even though the diagnosis of UTI was made in only 58% of patients. In a retrospective study of patients over 50 years of age with bacteremic UTI, the diagnosis of UTI was retained in only 36% of cases [23].

The mortality rate in our study, evaluated at 22% at 90 days, is comparable to the rates described in the literature for bacteremic UTI [19], but much lower than the 40% mortality rate at 90 days found in a recent study of older patients with all-cause bacteremia [9]. These data support previous reports that UTI-related bacteremia is associated with a better prognosis than bacteremia, secondary to other infections [34]. This may partly explain why, contrary to our expectations, we did not find an excess risk of mortality when antibiotics were not administered in the initial phase. This reinforces the idea that, outside emergency situations, practitioners can take the time of an appropriate diagnosis before starting antibiotic treatment. However, in a large observational study among 312,896 UTI outpatients, those who received delayed antibiotic treatment were at higher risk of bacteremia, hospitalization and all-cause mortality [35]. Conversely, initial diagnosis of UTI was associated with decreased mortality. This may be explained by the fact that atypical presentation of sepsis is associated with worse outcome in older patients [9], rather than the delay in antibiotic introduction.

In our series, the presence of UTI symptoms did not impact 90-day mortality. The current literature is inconsistent on this point [2,9,36]. Conversely, the absence of fever, reported in 40% of bacteremic patients [2,9], was associated with worse prognosis. This has already been described in the literature [1,7,34,37]. This higher mortality in apyretic patients can be explained by different hypotheses: the absence of hyperthermia may falsely reassure the clinician and induce a delay in the initiation of antibiotic therapy, but it may also reflect immunosenescence [37]. Like other authors, we found that the presence of acute urine retention on initial management is a risk factor for mortality. Urinary retention or the presence of post-void residual leads to urine stasis, which increases the risk of colonization and increases the septic risk after urological procedures [38], but these situations could also be a simple marker of comorbidity and fragility [39,40].

This study has several limitations. First, the interpretation of these results is limited by its retrospective design. Several biomarkers, including procalcitonin, have thus not been recorded. Moreover, UTI symptoms, especially in paucisymptomatic forms, may have been under-reported in the medical record. This study also only included patients for whom urinalysis and blood cultures have been prescribed, and such prescriptions are usually done in the presence of symptoms suggestive of infection. It is, therefore, plausible that a significant portion of patients with paucisymptomatic bacteremic UTI have not been diagnosed and were thus not included. However, this study illustrates the real-world situation. Secondly, this study included only bacteremic forms: clinical presentation and prognostic factors cannot be generalized to all UTIs. Thirdly, we chose to include only patients infected with GNB, which, although by far the majority, represent only a portion of uropathogens [5]. This choice guaranteed the urinary gateway to these infections, and thus excluded blood-borne UTIs, particularly staphylococcal [41], which have a different pathophysiology. Similarly, in hospitalized patients with candidemia, concomitant candiduria is usually an independent event [42]. Finally, the monocentric design and the small number of included patients are limitations, and certain prognostic factors may have been overlooked due to a lack of power.

However, this unselected real-world series is one of the first comprehensive reports of atypical clinical forms and prognostic factors in bacteremic UTI in the very old. More observational data from older patients are needed to better characterize the age-related clinical specificities of such a frequent condition and to establish the prognostic relevance of these potential signs.

## 5. Conclusions

In this unselected cohort of older inpatients with UTI-related bacteremia, UTI symptoms were found in only one-third of patients and an initial diagnosis of UTI was made in only 58%. The absence of UTI symptoms was not associated with a worse prognosis, but afebrile presentation, found in 40% of patients, was associated with an increased mortality. While the presence of bacteriuria is not sufficient for UTI diagnosis, these data are a reminder that typical UTI symptoms are absent in a majority of older patients. The diagnosis of UTI is, therefore, particularly difficult in geriatrics, but remains of significant prognostic interest, since appropriate early diagnosis appears to be associated with lower mortality.

## Figures and Tables

**Table 1:** Patients’ characteristics, clinical presentation, acute management and outcomes (*n* (%) or mean ± standard deviation).

	All Patients *n* = 105	UTI Signs *n* = 38	No UTI Signs *n* = 67	*p*
**Demographics**				
Age (*years*)	85.3 ± 5.9	85.1 ± 5.2	85.5 ± 6.3	0.76
Women	65 (61.9)	17 (44.7)	48 (71.6)	0.01
Nursing-Home Resident	30 (28.6)	11 (28.9)	19 (28.4)	0.95
ADL ≤ 4 (*n* = 86)	44 (51.2)	26 (49.1)	18 (54.5)	0.62
Chronic Urinary Catheter	19 (18.1)	11 (28.9)	8 (11.9)	0.03
**Acquisition of Infection**				
Community	52 (49.5)	23 (60.5)	29 (43.3)	0.09
Nursing-Home	27 (25.7)	9 (23.7)	18 (26.9)	0.72
Hospital	26 (24.8)	6 (15.8)	20 (29.9)	0.11
**Comorbidities**				
Charlson Comorbidity Index	62 (59)	20 (52.6)	42 (62.7)	0.31
Major Neurocognitive Disorder	30 (28.6)	10 (26.3)	20 (29.9)	0.70
Diabetes	27 (25.7)	6 (15.8)	21 (31.3)	0.08
Chronic Heart Failure	32 (30.5)	8 (21.1)	24 (35.8)	0.11
Stroke	18 (17.1)	6 (15.8)	12 (17.9)	0.78
Chronic Kidney Disease	16 (15.2)	6 (15.8)	10 (14.9)	0.91
Chronic Respiratory Disease	15 (14.3)	4 (10.5)	11 (16.4)	0.56
Neoplasia	21 (20)	11 (28.9)	10 (14.9)	0.36
Malnutrition	101 (96.2)	36 (94.7)	65 (97)	0.62
Number of Chronic Medications	7.4 ± 4.0	6.76 ± 3.4	7.78 ± 4.2	0.21
**Clinical Presentation**				
Sepsis	85 (81)	31 (81.6)	54 (8.6)	0.90
SBP ≤ 100 mmHg	32 (3.5)	8 (21.1)	24 (35.8)	0.11
Temperature ≥ 38.3 °C	61 (59.8)	24 (64.9)	37 (56.9)	0.43
Temperature ≤ 36.0 °C	3 (2.9)	1 (2.7)	2 (3.1)	1
Chills	25 (23.8)	8 (21.1)	17 (25.4)	0.62
qSOFA ≥ 2	29 (27.6)	8 (21.1)	21 (31.3)	0.26
**UTI Signs/Symptoms**	38 (36.2)	38 (100)	0	-
Acute Urine Retention	12 (11.4)	12 (31.6)	0	-
Hematuria	12 (11.4)	12 (31.6)	0	-
Urinary Burns	10 (9.5)	10 (26.3)	0	-
Suprapubic Pain	8 (7.6)	8 (21.1)	0	-
Acute Incontinence	6 (5.7)	6 (15.8)	0	-
Flank Pain	5 (4.8)	5 (13.2)	0	-
Pollakiuria	5 (4.8)	5 (13.2)	0	-
**Geriatric Symptoms**	44 (41.9)	12 (31.6)	32 (47.2)	0.11
Fall	21 (20)	4 (1.5)	17 (25.4)	0.08
ADL Degradation	17 (16.2)	6 (15.8)	11 (16.4)	0.93
**Biological Data**				
Leucocytes (*10^9^/l*)	14.1 ± 7.3	18 ± 7.0	14.7 ± 8.3	0.02
C Reactive Protein (*mg/L*)	144.5 ± 99.1	142.5 ± 113.4	145.5 ± 91.1	0.88
*E. coli*	82 (78.1)	31 (81.6)	51 (76.1)	0.52
**Initial Diagnosis**				
Sepsis	90 (85.7)	35 (92.1)	55 (82.1)	0.26
UTI	64 (58.1)	31 (81.6)	30 (44.8)	<0.001
Respiratory Tract Infection	20 (19)	2 (5.3)	18 (26.9)	0.008
**Antibiotic Treatment**				
Started Within 24 h	84 (80)	32 (84.2)	52 (77.6)	0.42
GNB Spectrum	73 (69.5)	31 (81.6)	42 (62.7)	0.04
**Outcomes**				
In-Hospital Mortality	15 (14.3)	6 (15.8)	9 (13.4)	0.74
90 Days Mortality (*n =* 89)	21 (23.6)	9 (29)	12 (20.7)	0.38

ADL: Activity of daily living scale; GNB: gram negative bacilli; qSOFA: quick Sequential Organ Failure Assessement; SBP: systolic blood pressure; UTI: urinary tract infection.

**Table 2:** Factors associated with 90-day mortality after bacteremic urinary tract infection (UTI).

	Bivariate Analysis HR [95% CI]	Multivariate Analysis HR [95% CI]
**Demographics**		
Age ≥ 85 Years	0.83 [0.35–1.96]	
Women	0.27 [0.11–0.66]	0.29 [0.10–0.79]
Nursing Home Resident	1.22 [0.49–3.03]	
ADL Scale ≤ 4	1.66 [0.56–4.96]	
Chronic Urinary Catheter	1.55 [0.57–4.23]	
**Acquisition of Infection**		
Community	0.65 [0.27–1.58]	
Nursing-Home	0.83 [0.30–2.26]	
Hospital	1.94 [0.80–4.68]	
**Comorbidities**		
Charlson Comorbidity Index ≥ 2	1.42 [0.57–3.52]	
Major Neurocognitive Disorder	0.52 [0.18–1.56]	
Number of Chronic Medications ≥ 5	0.93 [0.34–2.55]	
**Clinical Presentation**		
Sepsis	1.10 [0.37–3.26]	
Temperature ≥ 38.3 °C	0.39 [0.16–0.95]	0.37 [0.14–0.97]
qSOFA ≥ 2	3.51 [1.49–8.30]	
UTI Signs/Symptoms	1.42 [0.60–3.38]	
Acute Urine Retention	4.55 [1.75–11.8]	4.29 [1.41–13.05]
Lower Urinary Tract Symptoms	1.96 [0.83–4.66]	
**Biological Data**		
Leucocytes (*10^9^/l*)	0.99 [0.95–1.04]	
C Reactive Protein (*mg/L*)	1.00 [0.99–1.00]	
*E. Coli*	0.63 [0.21–1.88]	
Initial Diagnosis		
Sepsis	0.73 [0.25–2.18]	
UTI	0.41 [0.17–0.99]	0.35 [0.13–0.94]
**Antibiotic Treatment**		
Started within 24 h	1.08 [0.36–3.21]	
GNB Spectrum	1.37 [0.50–3.75]	

ADL: Activity of daily living scale; CI: Confidence interval; GNB: gram-negative bacilli; HR: hazard ratio; qSOFA: quick sequential organ failure assessement.

## Data Availability

Data supporting reported results are available from the corresponding author on reasonable request.
